# Is Switzerland Suitable for the Invasion of *Aedes albopictus*?

**DOI:** 10.1371/journal.pone.0082090

**Published:** 2013-12-13

**Authors:** Markus Neteler, Markus Metz, Duccio Rocchini, Annapaola Rizzoli, Eleonora Flacio, Luca Engeler, Valeria Guidi, Peter Lüthy, Mauro Tonolla

**Affiliations:** 1 Department of Biodiversity and Molecular Ecology, Research and Innovation Centre, Fondazione Edmund Mach, S. Michele all’Adige (TN), Italy; 2 Mosquito Working Group, Department of Health, Canton Tessin, Bellinzona, Switzerland; 3 Regional Laboratory for Biosafety, Institute of Microbiology, Canton Tessin, Bellinzona, Switzerland; 4 Institute of Microbiology, ETH Zurich, Zurich, Switzerland; 5 Microbial Ecology Group, Microbiology Unit, Plant Biology Department, University of Geneva, Geneva, Switzerland; Fondazione Bruno Kessler, Italy

## Abstract

**Background:**

Over the last 30 years, the Asian tiger mosquito, *Aedes albopictus*, has rapidly spread around the world. The European distribution comprises the Mediterranean basin with a first appearance in Switzerland in 2003. Early identification of the most suitable areas in Switzerland allowing progressive invasion by this species is considered crucial to suggest adequate surveillance and control plans.

**Methodology/Principal Findings:**

We identified the most suitable areas for invasion and establishment of *Ae. albopictus* in Switzerland. The potential distribution areas linked to the current climatic suitability were assessed using remotely sensed land surface temperature data recorded by the MODIS satellite sensors. Suitable areas for adult survival and overwintering of diapausing eggs were also identified for future climatic conditions, considering two different climate change scenarios (A1B, A2) for the periods 2020–2049 and 2045–2074. At present, the areas around Lake Geneva in western Switzerland provide suitable climatic conditions for *Ae. albopictus*. In northern Switzerland, parts of the Rhine valley, around Lake Constance, as well as the surroundings of Lake Neuchâtel, appear to be suitable for the survival at least of adult *Ae. albopictus.* However, these areas are characterized by winters currently being too cold for survival and development of diapausing eggs. In southern Switzerland, *Ae. albopictus* is already well-established, especially in the Canton of Ticino. For the years 2020–2049, the predicted possible spread of the tiger mosquito does not differ significantly from its potential current distribution. However, important expansions are obtained if the period is extended to the years 2045–2074, when *Ae. albopictus* may invade large new areas.

**Conclusions/Significance:**

Several parts of Switzerland provide suitable climatic conditions for invasion and establishment of *Ae. albopictus*. The current distribution and rapid spread in other European countries suggest that the tiger mosquito will colonize new areas in Switzerland in the near future.

## Introduction

*Aedes albopictus* (Skuse, 1984) (Diptera: Culicidae), the Asian tiger mosquito, is considered one of the most important invasive species world-wide [1]. In the last 30 years, it has been introduced to the USA, Europe, Africa, and the Indo-Pacific region [2] mainly through international trade of used tires and the import of lucky bamboo (*Dracaena sanderiana*). From the original points of entry *Ae. albopictus* has usually been spread passively by road traffic [1]. As an efficient vector of at least 26 arboviruses [2], including dengue, and Chikungunya, *Ae. albopictus* is considered a major risk to public health in Europe [1]. The tiger mosquito is firmly established in several countries bordering the Mediterranean, including Spain, France, Italy, Croatia and Greece [3]. Key populations of introduced *Ae. albopictus* exist in the Netherlands, especially in greenhouse nurseries which serve as points of entry for imports of lucky bamboo plants from Asia.

**Figure 1:**
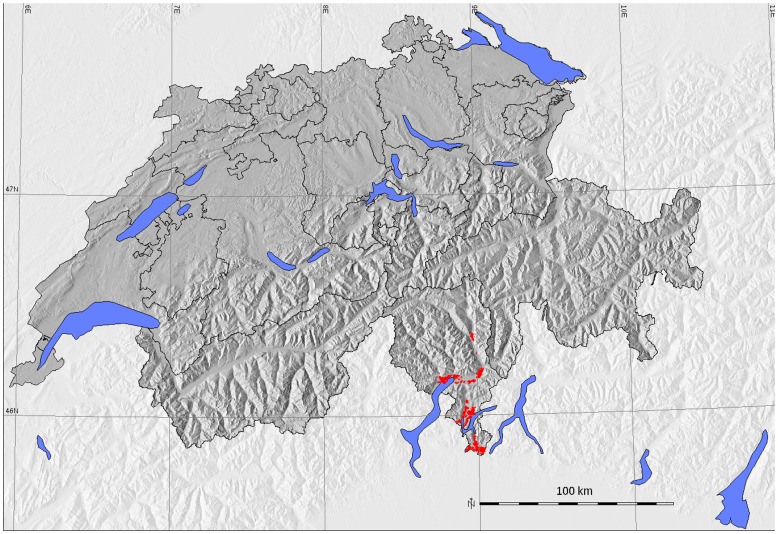
Territory of Switzerland with cantons. The distribution areas of *Ae. albopictus* from 2012 are shown in red.

*Ae. albopictus* was the vector responsible for a Chikungunya fever outbreak in the province of Ravenna (Italy) in 2007 [4] as well as single cases in France in 2010 [5]. Autochthonous cases of Dengue were registered in France and Croatia in 2010 [6], [7]. In Switzerland, *Ae. albopictus* was recorded for the first time in summer 2003, in the Canton of Ticino [8], [9] within a monitoring program started in 2000 by the local Mosquito Working Group (Gruppo Lavoro Zanzare, Canton of Ticino) [10]. Efforts to control and eliminate *Ae. albopictus* from the Swiss territory were unsuccessful. In 2007 it became firmly established in urban areas bordering Italy and started to expand towards northwards [10]. At the end of 2012, a total of 50 communities were reported to be infested by *Ae. albopictus* (Fig. 1).

Distribution models for *Ae. albopictus* predict a further expansion in European countries, backed-up by general globalization, and especially climatic changes, increasing public health risks [11], [3], [12], [13]. In order to early identify the most suitable control options for timely prevention and control of *Ae. albopictus*, it is necessary to identify and predict which are the most suitable areas for its potential introduction and establishment.

The spatial distribution and colonization of new areas by *Ae. albopictus* depend on several environmental parameters, such as winter and summer temperatures, and precipitation patterns. *Ae. albopictus* is known to be sensitive to low temperature [14], [11], [15], especially for overwintering. As shown previously [13], [16], [17], January mean temperature can be used as a threshold to estimate the survival chances of overwintering diapausing eggs, whereas the annual mean temperature can be used as a threshold to estimate population stability. The suitability of an area to the survival of *Ae. albopictu*s populations can also be determined by growing degree days (GDD) derived from temperature time series [16]. Growing degree days are defined as the degrees exceeding a given threshold (11°C for *Ae. albopictus*) accumulated for all days in a given year [14]. Furthermore, *Ae. albopictus* can not survive in arid areas; 500 mm precipitation per annum is considered the minimum threshold value [18], [19]. Many areas in Europe receive sufficient precipitation for *Ae. albopictus* to survive, including Switzerland, with mean annual precipitation ranging from 840 mm to 1,900 mm (European Climate Assessment and Dataset (ECA&D, http://www.ecad.eu/, [20]). Here we use the same environmental limits as established and used in previous studies [13], [14], [11], [16], [17]. For a detailed comparison of the different approaches refer to [21].

In order to predict the possible spread of the tiger mosquito it is crucial to consider the current climatic conditions and expected future scenarios. In fact, even the more optimistic climate scenarios predict that mean annual temperatures increasing over the next few decades across the country [22]. This predicted climatic change may lead to an increase in the spatial distribution of *Ae. albopictus*. The aim of this study was to identify the most suitable areas in Switzerland for the invasion and establishment of *Ae. albopictus* considering both current and future climate conditions. To assess the potential distribution areas, two different climate change scenarios for Switzerland for the periods 2020–2049 and 2045–2074 were considered.

## Materials and Methods

### Study Area

The study area comprises the entire territory of Switzerland (between 45° N and 48° N latitude, 5°E and 11°E longitude; see Fig. 1). The overall area of approximately 41,300 km^2^ is characterized by a great diversity of landscapes and climates. The country is topographically divided in tree main regions: the Jura Mountains, the Swiss Plateau and the Alps. The majority of the territory (60%) is occupied by the Alps, and the population (about 8 million people) lives mainly on the Swiss Plateau. In general, the Swiss climate can be considered as temperate, but it can vary considerably between regions because the Alps act as a climate barrier between northern and southern Switzerland. In addition, the Mediterranean Sea influences the climate of southern Switzerland, characterized by much milder winters than the northern part of the country.

### Data Sources for Current Conditions

A general problem in spatial modeling with environmental and meteorological variables is the uneven distribution of meteorological stations, which is particularly problematic for a complex terrain such as that characteristic for Switzerland. An alternative to meteorological stations is remote sensing which can obtain temperature time series with a high spatial resolution. A sensor especially designed for this kind of environmental monitoring is MODIS (Moderate Resolution Imaging Spectroradiometer) on board the satellites Terra and Aqua [16]. For MODIS on Terra, data are available from March 2000 onwards; for MODIS on Aqua, data are available from July 2002 onwards. Land Surface Temperature (LST) from MODIS is captured with four records per day, at approximately 1∶30 am, 10∶30 am, 1∶30 pm, and 10∶30 pm local time (Greenwich Mean Time +1∶00). After a short processing time of one week after image acquisition, the MODIS products are provided to the public free of charge. The spatial resolution of the MODIS LST products is 1,000 m, allowing for a detailed analysis on national and regional level. With a temporal resolution of 4 values per day and spatial resolution of 1000 m, this dataset is well suited for a spatialized risk assessment of *Ae. albopictus*. Gridded datasets derived from meteorological station records are usually only provided with a spatial resolution of 20 km or coarser [23], [20]; this spatial resolution is not adequate for topographically complex terrain such as found in Switzerland. With regard to temporal resolution, certain environmental indicators such as growing degree days can only be calculated with at least one data point per day. Since land surface temperature data obtained with remote sensing can contain gaps due to cloud cover, these gaps needed to be filled by reconstructing any missing LST values before environmental indicators can be derived from these data. LST reconstruction was achieved with a combination of statistical (principal component analysis, multiple regression analysis) and spline-based surface interpolation methods including auxiliary parameters related to land surface temperature (method based on [24]). For validation, LST values were compared with records from 1,487 meteorological stations (GSOD) for 240 days. The correlation between the LST values and the station record was very good with R^2^ = 0.78 averaged over all days. We also calculated the differences in monthly average temperature between GSOD records and MODIS LST data for the months January, April, July, October in the years 2003 and 2010. The spatial averages for Switzerland of the monthly differences ranged from −3.12°C to 3.78°C. The inclusion of elevation as one of the auxiliary parameters allowed an increase in the spatial resolution to 250 m. The final data set used in this study consists of four temperature maps per day from July 8, 2002 onward until end of 2012.

Average annual precipitation was calculated from the European Climate Assessment and Dataset (ECA&D, http://www.ecad.eu/, [20]) using the time series aggregation functions of GRASS GIS [25]. The original data resolution is 0.25 degrees. The ECA&D data show a current minimum annual precipitation of 840 mm/year (the lower boundary of the range obtained from long-term average annual precipitation within Switzerland) for the territory of Switzerland, which is higher than the minimum needed for the establishment of the tiger mosquito (i.e. 500 mm/year [18], [19]). Accordingly, precipitation values were not considered in our models.

### Data Sources for Future Conditions

Data on projected future temperature and precipitation regimes for Switzerland were obtained from the Swiss Climate Change Scenarios CH2011 (http://data.c2sm.ethz.ch/dataset/ch2011/seasonal_regional/download.html) [22], [26]. This data set is delivered as comma separated values (CSV) files with regional estimates (three regions: northeastern Switzerland (CHNE), western Switzerland (CHW), and Switzerland south of the Alps (CHS)) for projected future change for winter (DJF: December–February), spring (MAM: March–May), summer (JJA: June–August), and autumn (SON: September–November). The CH2011 data set consists of three parts: lower, medium and upper estimates of changes in temperature and precipitation relative to the reference period 1980–2009. Future conditions are available for three scenario periods (2020–2049, 2045–2074, 2070–2099), and the A1B, A2, and RCP3-PD emission scenarios. The A1B and A2 scenarios do not assume great efforts to limit or reduce climate change. The A1B scenario presumes a very rapid economic growth with the introduction of innovative and more efficient technologies, and equilibrium between fossil-intensive and no fossil energy sources. Depending on the season and region considered, the medium estimates of the A1B emission scenario foresee a warming of 0.9–1.4°C by the period 2020–2049, 2.0–2.9°C by 2045–2074, and 2.7–4.1°C by 2070–2099, as well as a decrease in summer mean precipitation by 10–17% by 2045–2074 and 18–24% by 2070–2099. The A2 emission scenario also includes an assumption about economic growth and technological progress, but at a slower rate and more heterogeneously. It predicts an increase in the seasonal mean temperature of 3.2–4.8°C by the end of the century (period 2070–2099) and a reduction in summer mean precipitations by 21–28%, depending on region. In contrast to A1B and A2 scenarios, the RCP3-PD emission scenario (peak and decline, also referred to as RCP2.6) presupposes the implementation of mitigation measures aiming at reducing the anthropogenic greenhouse gas emissions in the next decades, with a likely stabilization at an annual mean warming of 1.2–1.8°C and a summer drying of 8–10% by the end of the century (period 2070–2099) [22]. In this study, we focused on the well known A1B and A2 scenarios and did not consider the more aggressive mitigation scenario RCP3-PD following [27], [28].

In the present study, future suitable areas for *Ae. albopictus* adult survival and overwintering of diapausing eggs were assessed considering the A1B and A2 climate change scenarios for the period 2020–2049 (denoted by the corresponding central year 2035) and the period 2045–2074 (denoted by the corresponding central year 2060), using the seasonal and regional data tables for north-eastern, western, and southern Switzerland, respectively, following the spatial division of the Swiss Climate Change Scenarios (CH2011).

### Software

The explicit use of Free and Open Source Software (FOSS) conforms with the scientific principles of reproducibility and transparency [29], particularly for ecological and environmental modeling. For this project, spatial data processing was performed with the open source software packages GRASS GIS (http://grass.osgeo.org, GRASS GIS 7; [25]) and MRT (MODIS Reprojection Tool, https://lpdaac.usgs.gov/tools/modis_reprojection_tool, MRT 4.1). Statistical modeling was performed with R: A Language and Environment for Statistical Computing (http://www.R-project.org).

### *Aedes albopictus* Suitability

Temperature thresholds for *Ae. albopictus* survival and establishment were based on [14], [30] and [13]. As in [13], a gradient of suitability was used instead of a simplified yes/no classification. The use of a gradient also considers uncertainty in the temperatures as well as spatial uncertainty. The calculated gradients range from 0.0 (unsuitable) to 1.0 (highly suitable). Areas with suitability values between 0 and 1 are moderately suitable, and *Ae. albopictus* may or may not survive in these areas. We preferred to apply linear functions instead of a sigmoidal curve as used in [13] in order to avoid over-parametrization of the model. Fitting non-linear functions would suggest an accuracy higher than the one embedded in the data source, in particular for low-resolution grids where the actual temperature range within one pixel exceeds the upper and lower bounds of the curve fitting.

The threshold for survival probability of overwintering eggs in diapause was set to 1°C for mean January temperature with a margin of 2°C. Totally unsuitable conditions were thus defined as <−1°C, and totally suitable conditions as >3°C. Suitability maps ranging from 0.0 to 1.0 were created by setting all areas <−1°C to 0.0, all areas >3°C to 1.0, and areas with a mean temperature between −1° and 3°C were linearly scaled with (temperature +1.0)/4.0.

The threshold for survival probability of adults was set to 11°C for mean annual temperature with a margin of 2°C. Totally unsuitable conditions were thus defined as <9°C, and totally suitable conditions as >13°C. Suitability maps ranging from 0.0 to 1.0 were created by setting all areas <9°C to 0.0, all areas >13°C to 1.0, and areas with a mean temperature between 9°C and 13°C were linearly scaled with (temperature −9.0)/4.0.

**Figure 2:**
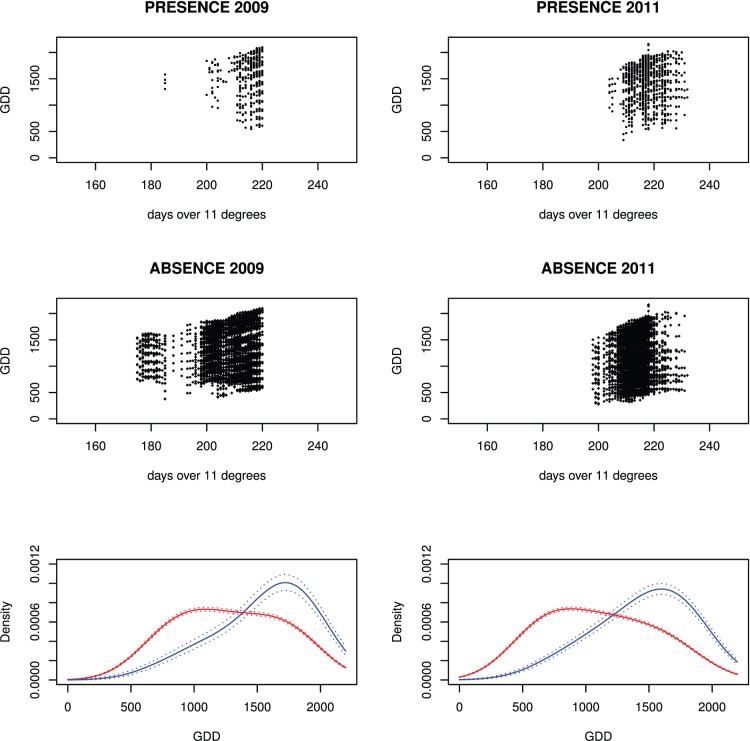
Growing Degree Days and number of days over 11°C at *Ae. albopictus* trapping sites. The number of Growing Degree Days (years 2009 and 2011) plotted against the number of days with at least 11°C at trapping sites with absence/presence of *Ae. albopictus* in Canton of Ticino, Switzerland. The density function plots combine the absence and presence data for the respective years.

The probability of *Ae. albopictus* completing its life cycle can be determined using Growing Degree Days (GDDs) [16]. The threshold for survival probability of adults based on 1,350 GDDs was set to 1st September, with a margin of one month. Totally unsuitable conditions were thus defined if 1,350 GDDs were reached only after day 274 of the year (October 1st), and totally suitable conditions if 1,350 GDDs were reached before the 214th day of the year (August 1st). Suitability maps ranging from 0.0 to 1.0 were created by setting all areas to 0.0 where 1,350 GDDs were reached after October 1st, all areas to 1.0 where 1,350 GDDs were reached before August 1st, and areas where 1,350 GDDs were reached between August 1st and October 1st were linearly scaled with (274 - day of year)/60. A statistical assessment using data for 2009 and 2011 (the year 2010 was unusually cold; pers. comm. Meteoswiss) following [14], [16] with trap data from Ticino confirmed that the threshold of 1,350 GDDs proposed in these studies is also appropriate for this canton. Figure 2 shows GDD plotted against the number of days over 11°C at the *Ae. albopictus* trapping sites in Canton Ticino, Switzerland (absence/presence of *Ae. albopictus*; years 2009 and 2011). For the years 2009 and 2011 the absence and presence data were combined to estimate a density function indicating the separation of absence and presence at the GDD threshold of 1,350 GDD as described in earlier publications.

The suitability analysis for the current conditions was performed for both the mean values over the years 2003–2011, and for 2011 only. Maps for adult suitability and overwintering suitability were also calculated using the A1B and A2 climate change scenarios for the period 2020–2049 and 2045–2074 on regional and seasonal level. The modeled deviation was then added to current conditions (the average over the years 2003 to 2011).

Overall suitability for current and future conditions was estimated by calculating the mean of the separate suitability indicators according to [13].

## Results and Discussion

The knowledge of a possible future spread of this mosquito species in areas not yet invaded and colonized is essential for implementing early prevention and control strategies.

Temperature is a significant parameter influencing the spatial distribution and population dynamics of *Ae. albopictus* [14], [15], especially in mountainous areas [31]. We generated potential distribution maps for *Ae. albopictus* considering the current climate conditions of Switzerland as well as the available future climate change projections.

**Figure 3:**
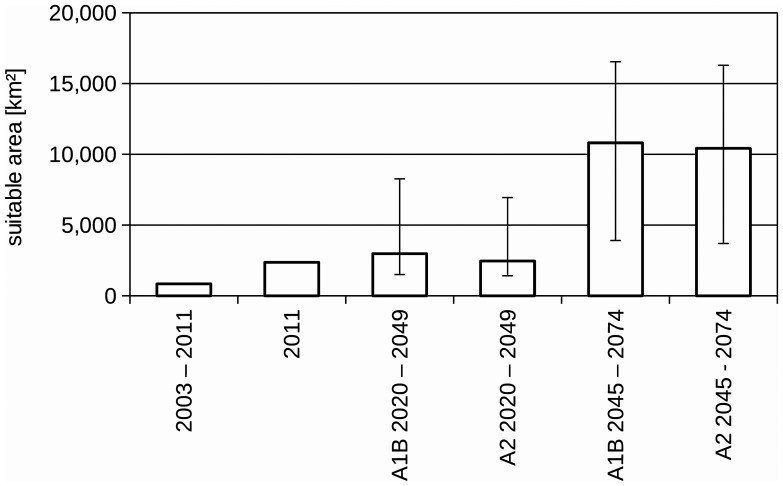
Amount of suitable area for *Ae. albopictus*. Amount of moderately to highly suitable (suitability > = 0.5) area for *Ae. albopictus* in Switzerland under current and future conditions. The error bars for the climate change scenarios represent the upper and lower estimates.

**Figure 4:**
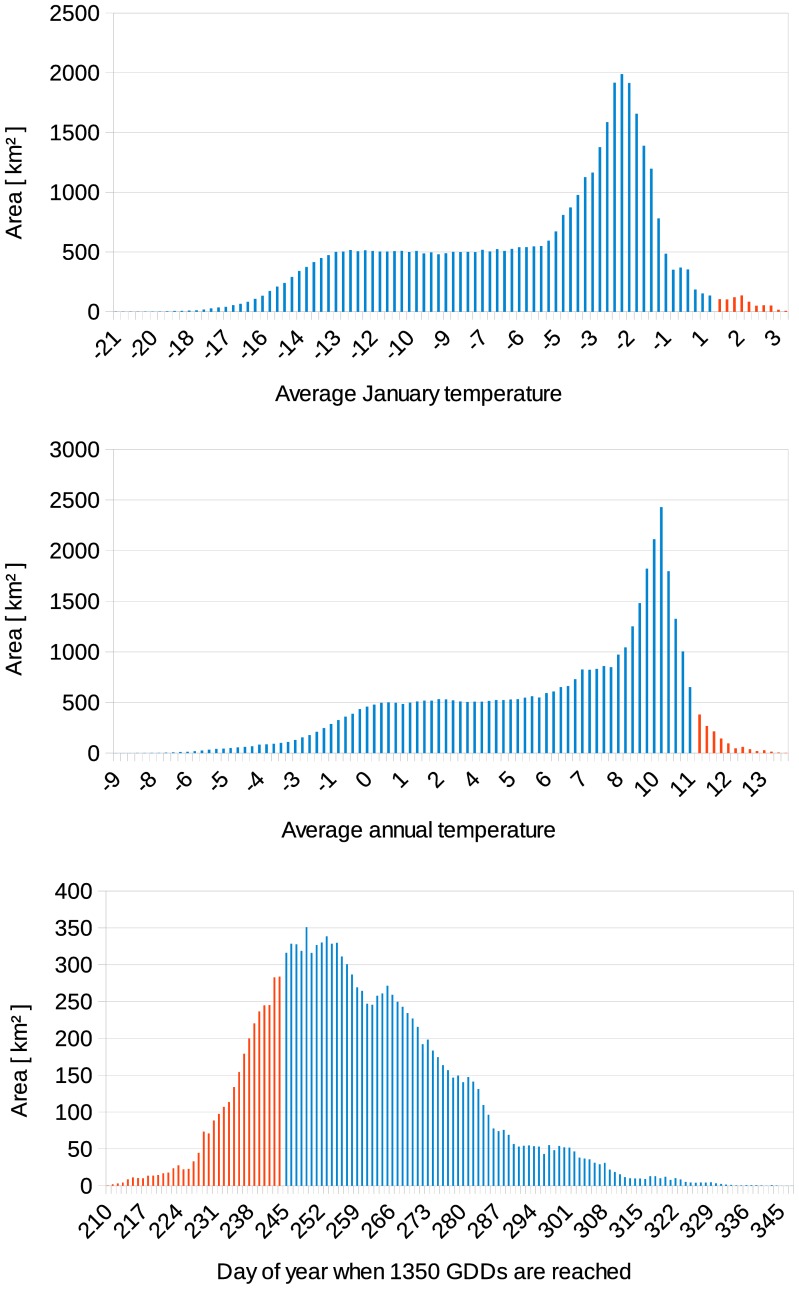
Sensitivity analysis of habitat model parameters. The histograms show the distribution of the model parameter values in Switzerland, based on the average for the period 2003–2011. Areas regarded as suitable for *Ae. albopictus* are in red, areas regarded as unsuitable are in blue. Small changes in the threshold value for the average January temperature would cause only small changes in the area regarded as suitable, whereas small changes in the average annual temperature and the day of year (DOY) when 1,350 GDDs are reached would result in much larger changes in the suitable areas.

Results obtained indicate that in the near future and based on our current knowledge on the bionomics of the species in alpine environment, the area suitable for the Asian tiger mosquito will expand considerably (Fig. 3), enabling the mosquito to invade northern regions of the country. Compared to 2003–2011, the size of the suitable area is estimated as three times as large for 2020–2049 and twelve times as large for 2045–2074. The modeling of habitat suitability is dependent on the specific thresholds for the used parameters January temperature, annual temperature, and day of year when 1,350 GDDs are reached (Fig. 4). Modifying the threshold for January temperate would only result in substantial changes if the threshold value would be lowered below −1°C. In contrast, lowering the threshold value for the annual temperature a few Kelvin would result in a substantial increase in the area regarded as suitable for this vector. Similarly, moving the threshold value when 1,350 GDDs are reached by about a week, also results in a substantial increase in the area regarded as suitable for the Asian tiger mosquito.

**Figure 5:**
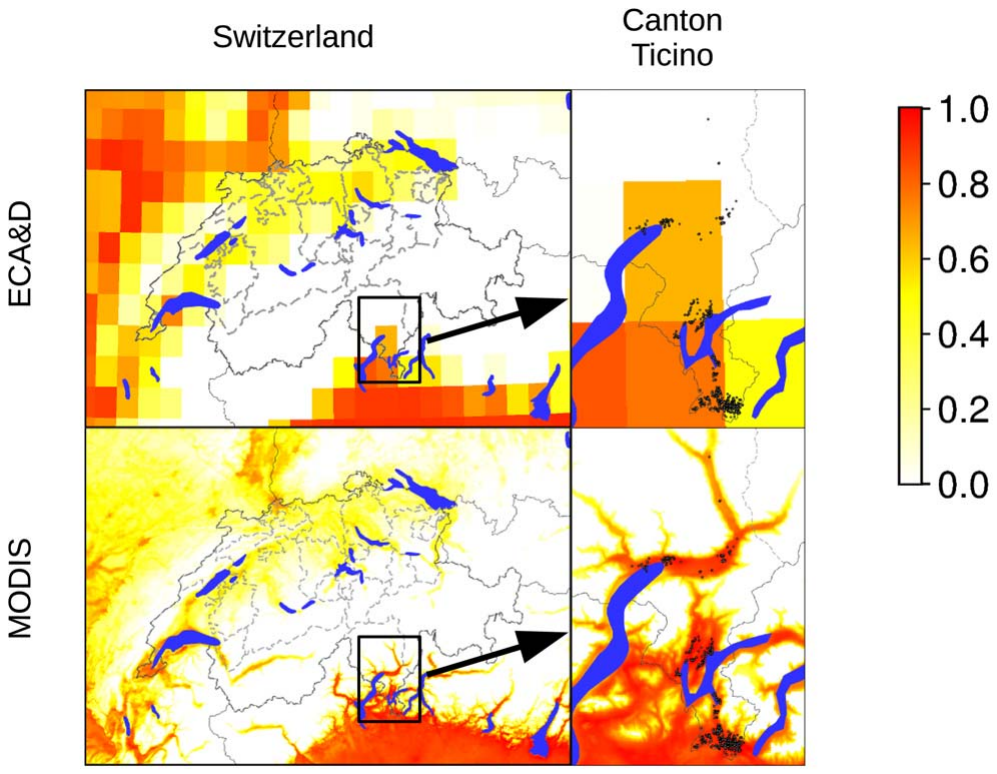
Comparison of models for current habitat suitability. In contrast to previously published approaches based on ECA&D, high resolution temperature data allow for the correct detection of areas suitable for *Ae. albopictus* in valleys. Dots in the maps for Canton Ticino indicate known presence of *Ae. albopictus*.

**Figure 6:**
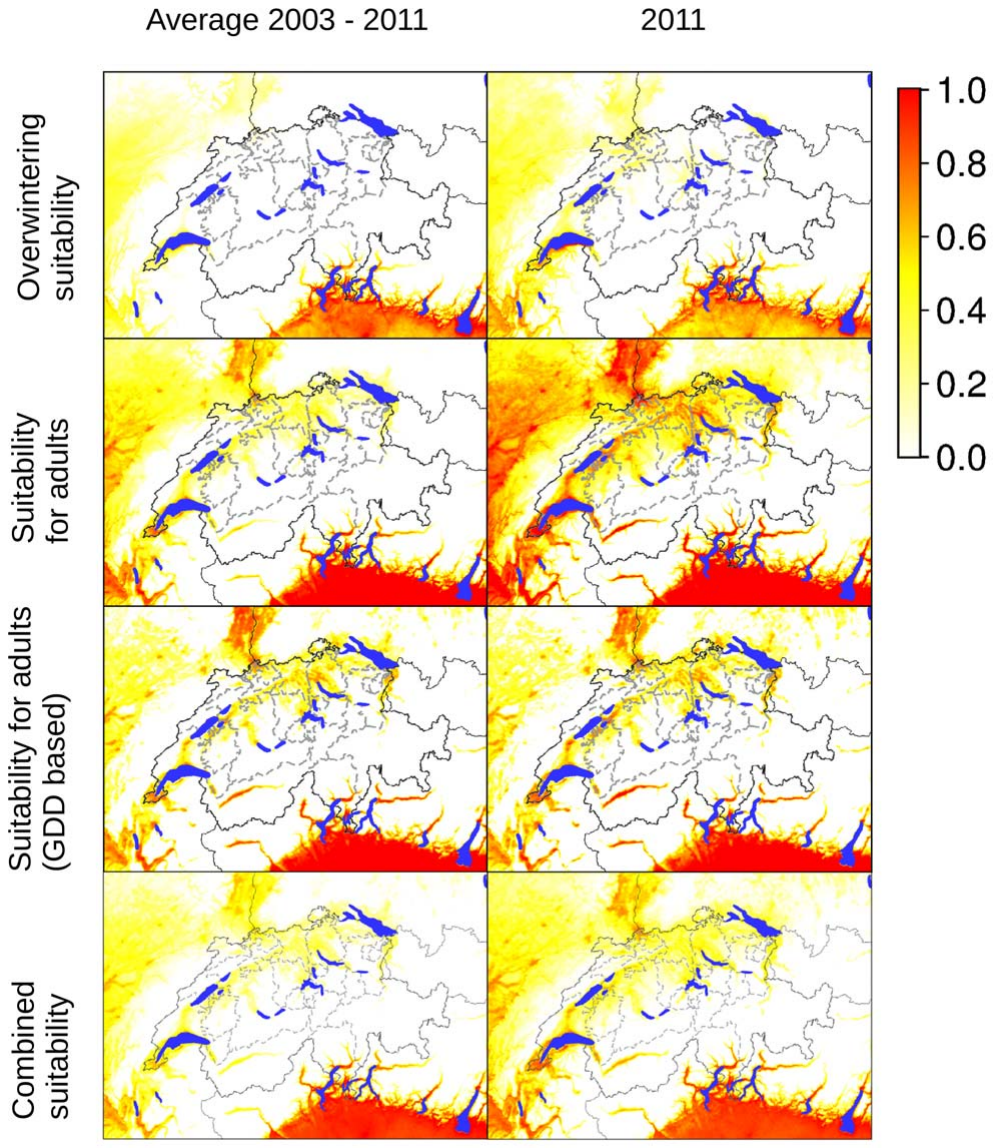
Suitability indicators for *Ae. albopictus* in Switzerland. The maps in the first column are based on averages for the years 2003–2011; the maps in the second column are based on temperature values for 2011 only. The coloring scheme shows the gradient of suitability, from 0.0 (unsuitable areas: white colored) to 1.0 (highly suitable areas: red colored). GDD: growing degree days filtered for days above 11°C.

The ability of diapausing eggs to survive the winter season is crucial for the establishment of new populations, allowing the populations’ persistence from one season to the next. This overwintering ability is related to the January mean temperature [32]. Considering the average temperature from 2003 to 2011, areas where *Ae. albopictus* encounters conditions for overwintering are the southern and central parts of the Canton of Ticino (Fig. 5), as well as areas near Lake Geneva (Fig. 6). Considering only temperatures of the year 2011, the potential distribution areas are wider including also marginally suitable sites within the Swiss Plateau (Fig. 6). The inability of diapausing eggs to survive the winter season in some regions, as the Rhône valley in the Wallis, reduces the risk of an establishment of tiger mosquito. This is consistent with other studies indicating that the dominant limiting factor for the survival and establishment of *Ae. albopictus* is the egg survival which is determined by January mean temperatures [16], [17]. However, our predictions are currently based on available estimates of temperature thresholds. More detailed estimates based on field observation and laboratory experiments carried out under controlled conditions should be available soon (A. Rizzoli, pers comm.).

Adult survival is mainly determined by mean annual temperatures, with 11°C as the lower limit for population establishment [14]. Our results show that suitable areas for adult survival and for successful life cycle completion in Switzerland are less restricted than the overwintering areas. In northern Switzerland, parts of the Rhine valley, both upstream and downstream of Lake Constance, as well as areas surrounding Lake Neuchâtel appear to be suitable for adults of *Ae. albopictus*, but are currently too cold in winter for the survival of eggs. Highly suitable areas for adult mosquitoes are the Canton of Geneva and areas surrounding Lake Léman, the main Rhône valley in the Canton of Wallis and the Canton of Ticino including the entire southern part, the region of Locarno, and the district of Riviera (Fig. 6).

**Figure 7:**
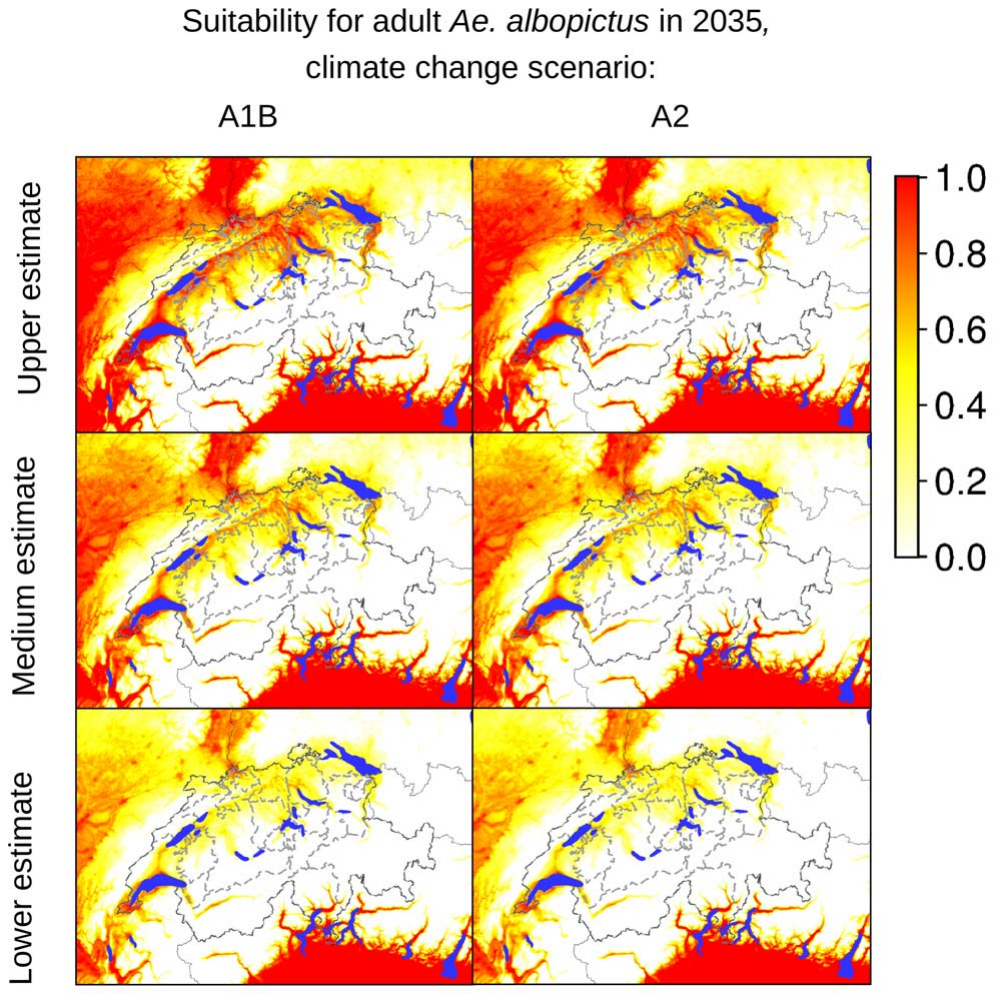
Suitability indicators for adult *Ae. albopictus* in Switzerland in 2035 (2020–2049). The maps in the first column are based on temperature estimates for the climate changes scenario A1B, the maps in the second column are based on temperature estimates for the climate changes scenario A2. The coloring scheme shows the gradient of suitability, from 0.0 (unsuitable areas: white colored) to 1.0 (highly suitable areas: red colored).

**Figure 8:**
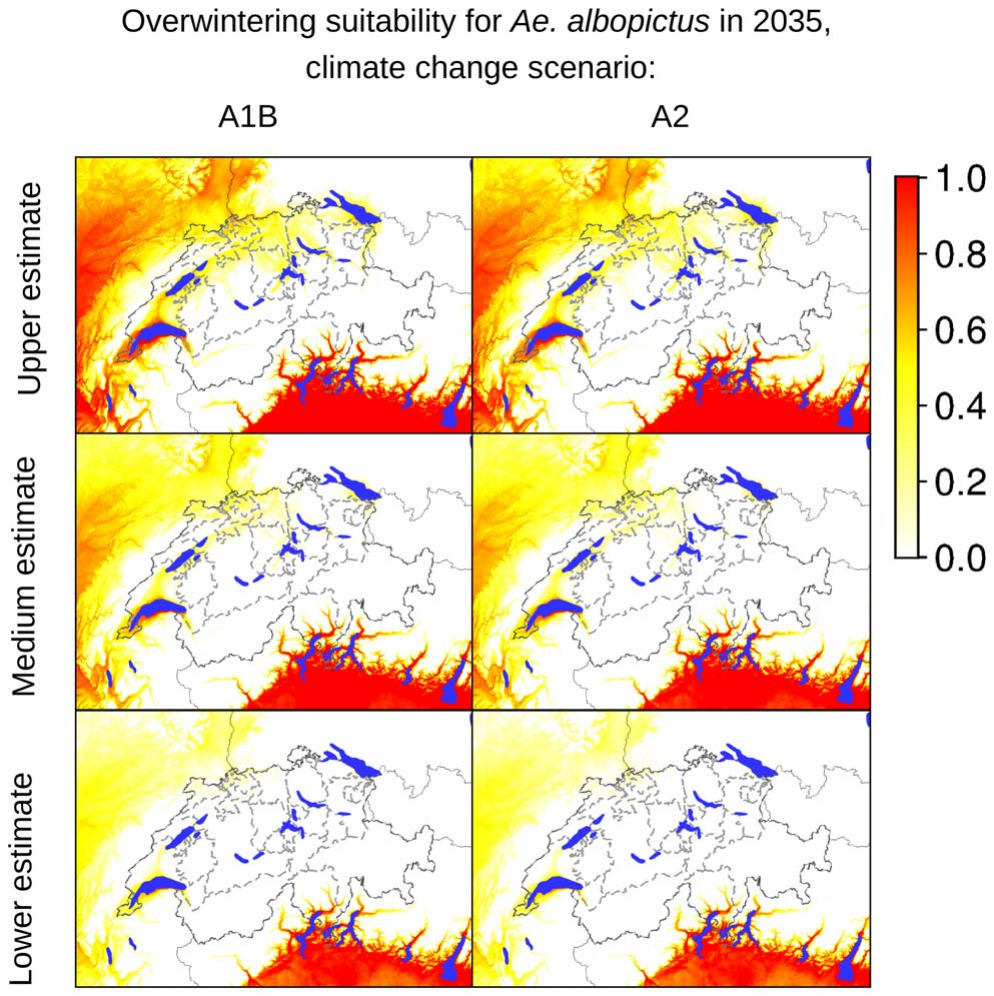
Suitability indicators for overwintering of *Ae. albopictus* in Switzerland in 2035 (2020–2049). The maps in the first column are based on temperature estimates for the climate changes scenario A1B, the maps in the second column are based on temperature estimates for the climate changes scenario A2. The coloring scheme shows the gradient of suitability, from 0.0 (unsuitable areas: white colored) to 1.0 (highly suitable areas: red colored).

**Figure 9:**
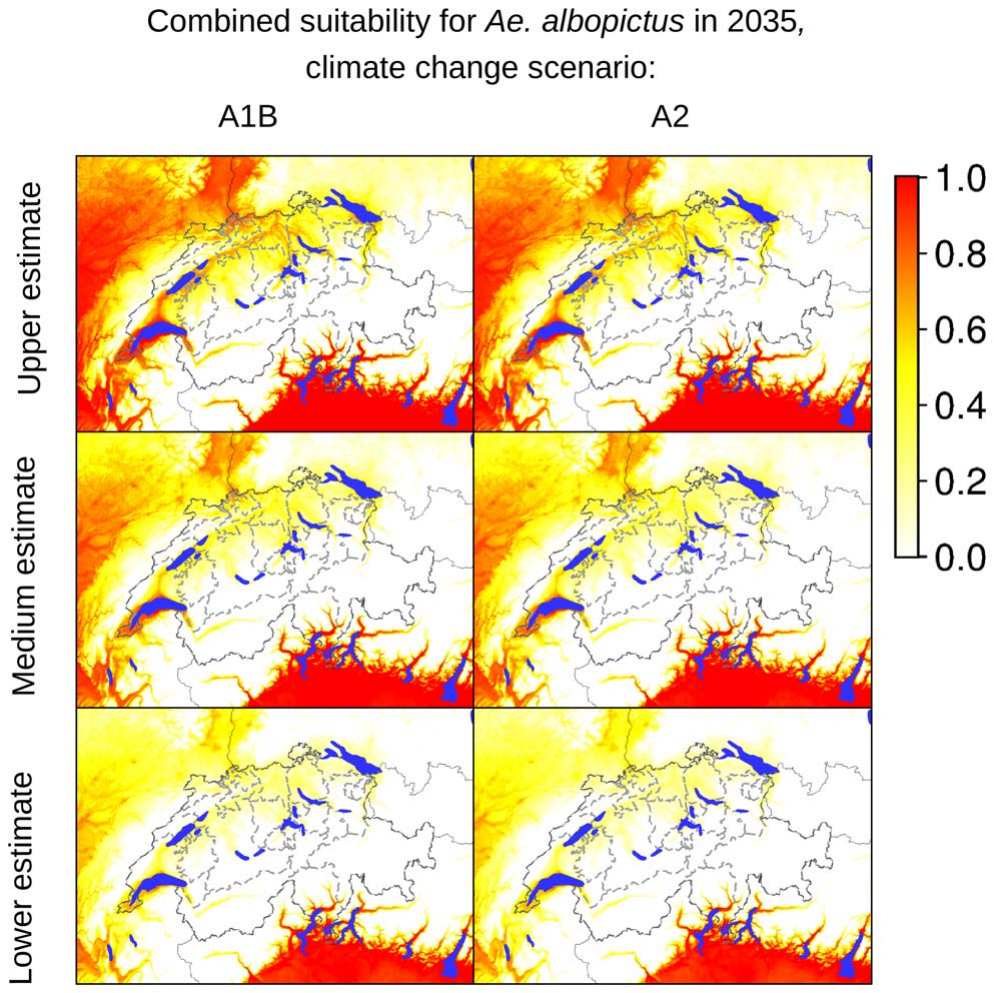
Combined suitability indicators for *Ae. albopictus* in Switzerland in 2035 (2020–2049). The maps in the first column are based on temperature estimates for the climate changes scenario A1B, the maps in the second column are based on temperature estimates for the climate changes scenario A2. The coloring scheme shows the gradient of suitability, from 0.0 (unsuitable areas: white colored) to 1.0 (highly suitable areas: red colored).

**Figure 10:**
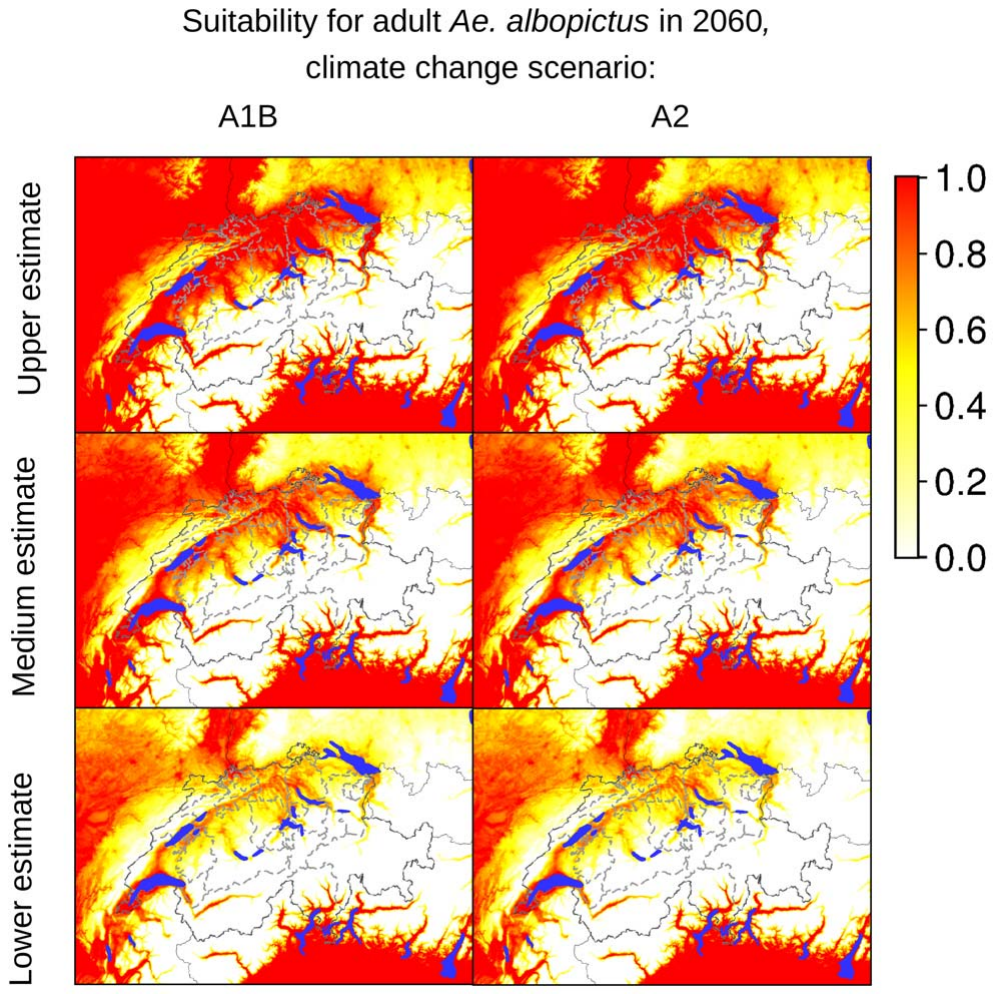
Suitability indicators for adult *Ae. albopictus* in Switzerland in 2060 (2045–2074). The maps in the first column are based on temperature estimates for the climate changes scenario A1B, the maps in the second column are based on temperature estimates for the climate changes scenario A2. The coloring scheme shows the gradient of suitability, from 0.0 (unsuitable areas: white colored) to 1.0 (highly suitable areas: red colored).

**Figure 11:**
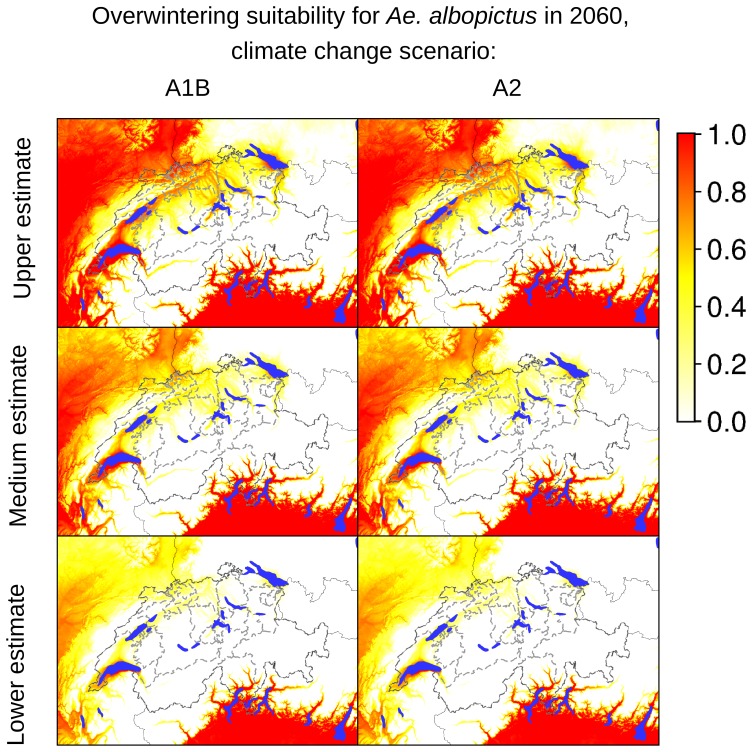
Suitability indicators for overwintering of *Ae. albopictus* in Switzerland in 2060 (2045–2074). The maps in the first column are based on temperature estimates for the climate changes scenario A1B, the maps in the second column are based on temperature estimates for the climate changes scenario A2. The coloring scheme shows the gradient of suitability, from 0.0 (unsuitable areas: white colored) to 1.0 (highly suitable areas: red colored).

**Figure 12:**
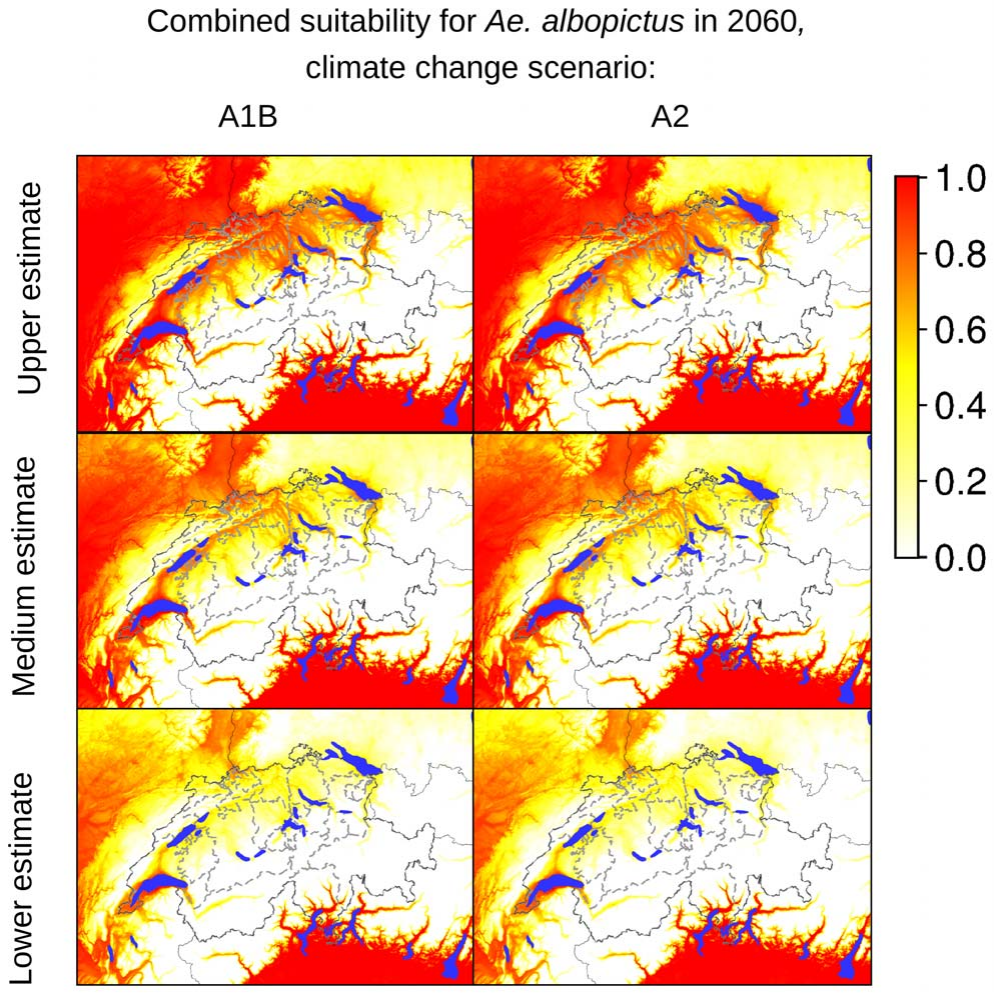
Combined suitability indicators for *Ae. albopictus* in Switzerland in 2060 (2045–2074). The maps in the first column are based on temperature estimates for the climate changes scenario A1B, the maps in the second column are based on temperature estimates for the climate changes scenario A2. The coloring scheme shows the gradient of suitability, from 0.0 (unsuitable areas: white colored) to 1.0 (highly suitable areas: red colored).

According to our analyses of the potential expansion of *Ae. albopictus* considering the future Swiss climate scenarios, the areas identified as currently suitable for *Ae. albopictus* will expand, as presented in Figs. 7–12, which show the simulated lower, medium and upper estimates of changes in temperature. The predicted decline of precipitation (A1B and A2 climatic scenarios) of up to 20% still does not drop below 500 mm/year, the threshold under which the establishment of the tiger mosquito is prevented. The ECA&D data show a current minimum of annual precipitation of 840 mm/year for Switzerland. The temperature data show low to significant differences for future scenarios compared to the present day. For the years 2020–2049, the predicted possible spread of the tiger mosquito does not differ significantly from its potential current distribution (Figs. 3, 7, 8 and 9). However, important expansions are obtained if the period is extended to the years 2045–2074, when *Ae. albopictus* may invade large new areas (2003–2011∶840 km^2^; A1B: 10,810 km^2^; A2∶10,420 km^2^, see also Fig. 4). Highly suitable regions for adult survival in the period 2045–2074 will comprise the whole Swiss territory, except the Alpine regions, i.e. central Switzerland, lateral Wallis valleys, and the Canton of Graubünden (Fig. 10). However, a part from the Canton of Ticino and areas surrounding Lake Geneva, regions highly suitable for adult survival remain only marginally or moderately suitable for the overwintering of eggs (Fig. 11). The overwintering suitability is predicted to increase substantially in neighboring countries, particularly in France. These areas may in the future act as a source for further invasion of *Ae. albopictus*. In western Switzerland, the areas around Lake Geneva could be invaded soon from France through mosquitoes especially imported through road transport. Neighboring regions in Italy, where *Ae. albopictus* is widely distributed [33], also provide sources for the continuous introduction of this invasive species to southern Switzerland (Bernasconi 2010, pers. comm.). A major traffic axis of international transport connects Italy to the Canton of Ticino and hence to northern Switzerland. *Ae. albopictus* may thus reach northern areas of Switzerland along this important traffic artery. For example, adult specimens of *Ae. albopictus* were recently trapped in the Upper Rhine Valley in south-west Germany, in a rest area along the A5 highway entering Germany from Switzerland [34], [35].

Some areas suitable for *Ae. albopictus* are at the same time characterized by a high density of humans, being relevant for the probability of observing an outbreak of diseases transmitted by *Ae. albopictus*. The canton Ticino has a mean population density of 119.8 humans/km^2^; the majority of the population resides in areas where the vector is established with the district of Mendrisio, near the Italian border, being the area with high density of mosquitoes and of human population (485.9 humans/km^2^). The canton of Geneva, an area where the mosquito is not yet established but which is at major risk of establishment, has a human population density of 1,633 humans/km^2^ (base year 2012; source: http://www.ti.ch/ustat).

The areas where *Ae. albopictus* is currently present in Switzerland (Fig. 1) are in line or in agreement with the highly suitable areas predicted by our models using current climate and environmental variables. Other studies modeling the possible spatial distribution of *Ae. albopictus* in Europe show similar data for Switzerland [12], [13] despite the lower resolution compared to our models (Fig. 5). This is the first time that an accurate distribution model for *Ae. albopictus* has been established for Switzerland. High resolution distribution models based on LST data from satellites have also been successfully created for the tiger mosquito for the Province of Trento and other north-eastern regions of Italy [17], [16].

In conclusion, various parts of Switzerland offer or will offer climatic conditions suitable for *Ae. albopictus*. Even though many potentially suitable areas are not yet invaded, the current distribution in Europe and the spread patterns suggests that the tiger mosquito might well appear in these areas within the next few years.
