# Correction: Is Switzerland Suitable for the Invasion of*Aedes albopictus*?

**DOI:** 10.1371/annotation/d1d46d6e-8152-4eab-8df9-7f2e5d14f391

**Published:** 2013-12-30

**Authors:** Markus Neteler, Markus Metz, Duccio Rocchini, Annapaola Rizzoli, Eleonora Flacio, Luca Engeler, Valeria Guidi, Peter Lüthy, Mauro Tonolla

The title of the article is incorrect. The correct title is: "Is Switzerland Suitable for the Invasion of *Aedes albopictus*?"

The correct citation is: Neteler M, Metz M, Rocchini D, Rizzoli A, Flacio E, et al. (2013) Is Switzerland Suitable for the Invasion of *Aedes albopictus*? PLoS ONE 8(12): e82090. doi:10.1371/journal.pone.0082090.

